# Tectorigenin targets PKACα to promote GLUT4 expression in skeletal muscle and improve insulin resistance *in vitro* and *in vivo*

**DOI:** 10.7150/ijbs.80125

**Published:** 2023-03-05

**Authors:** Xinlei Yao, Lei Liu, Wenjun Shao, Miao Bai, Xiaohan Ding, Geng Wang, Shuyue Wang, Lihua Zheng, Ying Sun, Guannan Wang, Yanxin Huang, Chunlei Yu, Zhenbo Song, Yongli Bao, Shaonian Yang, Luguo Sun

**Affiliations:** 1 National Engineering Laboratory for Druggable Gene and Protein Screening, Northeast Normal University, Changchun 130024, China.; 2 Key Laboratory of Neuroregeneration of Jiangsu and Ministry of Education, Co-innovation Center of Neuroregeneration, Nantong University, Nantong, China.; 3 Research Center of Agriculture and Medicine gene Engineering of Ministry of Education, Northeast Normal University, Changchun 130024, China.; 4 The Rolf Luft Research Center for Diabetes and Endocrinology, Karolinska Institutet, Karolinska University Hospital L1, Stockholm, Sweden.

**Keywords:** tectorigenin, insulin resistance, GLUT4, AMPK, PKACα

## Abstract

The decreased expression and dysfunction of glucose transporter 4 (GLUT4), the insulin-responsive glucose transporter, are closely related to the occurrence of insulin resistance (IR). To improve the expression of GLUT4 may represent a promising strategy to prevent and treat IR and type 2 diabetes (T2DM). Here, we demonstrate that the natural compound tectorigenin (TG) enhances GLUT4 expression, glucose uptake and insulin responsiveness via activating AMP-activated protein kinase (AMPK)/myocyte enhancer factor 2 (MEF2) signaling in both normal and IR skeletal muscle cells and tissues. Accordingly, prophylactic and therapeutic uses of TG can significantly ameliorate IR and hyperglycemia in T2DM mice. Mechanistically, we identify protein kinase A catalytic subunit α (PKACα) as the target of TG to increase GLUT4 expression and TG-PKACα binding promotes the dissociation of PKACα from the regulatory subunits, leading to the activation of PKA/AMPK signaling. PKACα knockdown in local quadriceps muscles almost completely abolished the therapeutic effects of TG on IR and T2DM, as well as the enhancement on AMPK signaling and GLUT4 expression in skeletal muscle. This study supports TG as a new drug candidate to treat IR and its related diseases, but also enriches our knowledge of PKA signaling in glucose metabolism in skeletal muscle.

## Introduction

Type 2 diabetes mellitus (T2DM) can lead to multiple organ damage and even be life-threatening 1. Insulin resistance (IR) is the core mechanism of T2DM, usually manifested by decreased insulin-stimulated glucose uptake and results from impaired insulin signaling and multiple post-receptor intracellular defects, including impaired glucose transport 2. Glucose transporter protein 4 (GLUT4) is the major insulin-regulated glucose transporter and its expression is mainly limited to insulin-responsive tissues, in which, insulin rapidly induces GLUT4 to translocate from the cytoplasm to the surface of the cell membrane to take up glucose for metabolism 3. In patients with IR, a decreased expression and abnormal translocation of GLUT4 are often observed, indicating that GLUT4 expression decrease is one of the important molecular mechanisms of IR 4, 5. Therefore, strategies focusing on enhancing GLUT4 function and expression represent a promising area to develop more effective drugs for the treatment of T2DM and other IR-related diseases.

Insulin-mediated activation of PI3K/AKT signaling through the insulin receptors is responsible for the induction of GLUT4 translocation from the cytoplasm to the cell membrane 6. Therefore, enhancing insulin sensitivity—for example, through the use of an insulin sensitizing agent—can promote glucose uptake by GLUT4. However, in accordance with the old saying, “you can't make bricks without straw”, if the GLUT4 expression level dramatically decreases in insulin-responsive tissues in T2DM patients, there is not enough GLUT4 to take up glucose even if the tissues respond well to insulin. Thus, the improvement of GLUT4 expression is no doubt essential for the achievement of overall amelioration in cases of IR. The promoter of *GLUT4* gene contains several responsive elements to transcription factors, among which myocyte enhancer factor 2 (MEF2) plays a central role in GLUT4 transcription. To date, several signaling pathways, such as AMP-activated protein kinase (AMPK) and calmodulin-dependent protein kinase (CaMK) signaling pathways, have been reported to be involved in regulating the expression of GLUT4 7. AMPK is an important energy-sensing protein kinase that is activated by the phosphorylation of threonine 172 (Thr172) within its catalytic α subunit in states of low cellular energy, which acts to restore an energy balance 8. AMPK regulates glucose transport, lipid and protein synthesis and other factors that have been linked to IR 9. It has been reported that AMPK signaling is involved in GLUT4 expression via the activation of MEF2A 10. Although the GLUT4 expression level is directly related to the glucose uptake capacity of insulin target tissues, researchers have not confirmed whether agents that are able to enhance GLUT4 expression would possess therapeutic effects on IR or T2DM. Moreover, there are no anti-T2DM or anti-IR drugs currently available in clinical practice with the pharmacological activity to increase GLUT4 levels. Therefore, we established a firefly luciferase reporter screening system driven by the *GLUT4* gene promoter to screen for potential compounds promoting GLUT4 expression and further investigated the effects of the compound candidates on IR or T2DM, thus exploring the possibility of obtaining potential anti-IR or anti-T2DM lead compounds using this screening system with GLUT4 as the target.

Tectorigenin (TG), an O-methylated isoflavone in *Belamcanda chinensis*, is one of the candidates that was screened using the above screening system. Interestingly, we previously revealed the preventive and therapeutic effects of TG on T2DM mice, as well as its protective effects on islet β-cells against glucotoxicity and lipotoxicity 11. TG has been shown to have endothelial anti-IR activity 12 and improve hyperglycemia and hyperlipidemia by regulating adipogenic differentiation and adipocytokines secretion 13. However, to our knowledge, the effects of TG on GLUT4 expression 13 have rarely been reported and especially the pharmacological target and working mechanisms underlying the anti-IR activity of TG, have not been fully elucidated.

Protein kinase A (PKA) is a widely expressed serine/threonine kinase that plays an important role in metabolism regulation. The PKA holoenzyme consists of two regulatory and two catalytic subunits. Upon activation, such as in the case of cAMP binding, the catalytic subunits are separated from regulatory subunits in order to phosphorylate downstream target proteins 14. PKA is an upstream kinase of the AMPK pathway 15 and recent research has shown that PKA activity was decreased in diet-induced obese rats 14. However, as far as we know, an effect of the regulation of PKA on GLUT4 expression has not been reported and no agonists of PKA have been utilized in the clinic to treat IR and T2DM so far. Here, we show that TG targets the catalytic subunit α of PKA (PKACα) to promote GLUT4 expression in muscles via activating the AMPK/MEF2 pathway, the main mechanism by which TG elicits its therapeutic effects on IR and T2DM. Our study provides a theoretical basis not only for developing new anti-IR or anti-T2DM drugs with TG as a lead compound but also for using PKA as an important target for IR or T2DM treatment.

## Methods

### Materials and reagents

TG (HPLC ≥ 98%) was purchased from Chengdu Biopurify Phytochemicals Ltd. (Chengdu, Sichuan, China). TG was dissolved in DMSO at a storage concentration of 10mg/ml for cell experiments. PA (catalog number P5585), Compound C (catalog number P5499) and H89 (catalog number 371963) were acquired from Sigma-Aldrich. *GLUT4* promoter-dependent luciferase reporter plasmid (pGL3-GLUT4-luc) was constructed in our laboratory.

### Cell culture

C2C12 cells were gifts from Dr. Zhenguo Wu (HKUST). C2C12 cells (within 15 passages) were cultured in Iscove's DMDM (CORNING, USA) containing 20% fetal bovine serum (GIBCO, USA). To induce cell differentiation, C2C12 cells were cultured to 100% fusion, and then cultured in IMDM containing 2% horse serum for 5-7 days to promote cell fusion into multinuclear myotubes. C2C12 myotubes were treated with 0.4 mM of palmitic acid (PA) for 24 h to induce insulin resistant cells. All of the cells were maintained under standard conditions (in a 37 ℃ incubator with 5% CO_2_) in the presence of 200 U/mL gentamycin sulfate.

### Luciferase assay

C2C12 cells were transfected with pGL3-basic, pGL3-GLUT4-luc or 3MEF2-luc 16 plasmids using lipofectamine 2000 reagent (Invitrogen) following the manufacturer's instructions. The pGL3-GLUT4-luc reporter plasmid was constructed by inserting the *GLUT4* gene promoter region (-1886~+43, obtained via PCR, primer sequences were listed in Table 1) upstream of the *Luciferase* reporter gene of the pGL3-basic plasmid. Transfected cells were treated with 10 μg/mL TG or not for 24 h and lysed for luciferase assays using a FLUOstar OPTIMA system (BMG Labtech, Offenburg, Germany) as previously described 17. Values shown represent the mean ± SEM from three separate experiments.

### Quantitative RT-PCR assays (RT-qPCR)

Quantitative RT-PCR was performed as previously described 11. Briefly, differentiated C2C12 cells were treated with 10 µg/mL TG for 24 h. The total RNA was extracted with TRIzol (Invitrogen) reagent and reverse-transcribed into cDNA. RT-qPCR was performed using a 2×FastStart Universal SYBR GreenMaster (Roche Diagnostics, Mannheim, Germany) system according to the manufacturer's protocol. The PCR primer sequences were listed in Table 1. Relative quantification of the expression of each gene was calculated using the ΔΔCT method.

### Western blotting (WB)

Total proteins were extracted from cells or homogenized tissues and subjected to Western blotting using the indicated antibodies as previously described 11. Anti-GAPDH (catalog number KC-5G4) antibodies were from Proteintech (Wuhan, Hubei, China). Antibodies against AKT (catalog number 4691), p-AKT (catalog number 4060), AMPK (catalog number 5832), p-AMPK (catalog number 2535) were from Cell Signaling Technology (Danvers, MA); Anti-GLUT4 (catalog number sc-53566), anti-PKAR (catalog number sc-136231), anti-PKACα (catalog number sc-28315) antibodies were from Santa Cruz Bio-technology (Santa Cruz, CA).

### siRNA transfection

SiRNAs (80 nM) were transfected into 50% confluent C2C12 cells using lipofectamine 2000 reagent (Invitrogen) following the manufacturer's instructions. The siRNAs were synthesized by Genepharma (Shanghai, China) and their sequences were listed in Table 2.

### Lentivirus Mediating PKACα Knockdown

The recombinant lentivirus encoding PKACα-shRNA (Lv-shPKACα), together with the Lv-shNC control lentivirus was purchased from Gene Pharma Co., Ltd. (Shanghai, China). The core sequences were listed in Table 2. To knock down PKACα in C2C12 cells, the Lv-shPKACα lentivirus was directly added to the C2C12 cells, and C2C12 cells cultured for infection with Lv-shNC were used as a negative control. To knock down PKACα in mouse muscle, see the section entitled *Animal Experiments*. The lentivirus-mediated knockdown of PKACα was confirmed via Western blotting.

### Molecular docking

PKACα (PDB: 5UZK) was selected as the docking target and TG (CID: 5281811) was selected as the ligand. All the water molecules in PKACα were removed and hydrogen atoms were added. The active site of PKACα was defined as those residues within 7 Å of the ligand in the X-ray structures. The default values of the parameters of the genetic algorithm were used, except that the ligand was subjected to 30 genetic algorithm runs, and the early termination option was turned off. GOLD 5.2 was adopted for molecular docking as previously described 18.

### Molecular dynamics simulation

The amber18 software package was used to perform molecular dynamics simulation experiments on the PKACα-TG complex obtained through molecular docking. For the protein PKACα, we used the ff14SB force field parameters, and for the small molecule TG, we used the GAFF general force field parameters. Based on these parameters, AM1-BCC atomic charges were obtained via the ANTECHAMBER module. In addition, we selected the TIP3P explicit water model. The workflows for molecular dynamics simulations were as follows. Firstly, we confined the heavy atoms of the protein PKACα (and the small molecule TG), and equilibrated the water with a 5000-step steepest descent method and a 5000-step conjugate gradient method to minimize the energy. Then, we slowly heated the system to 300 K in 50 ps. After heating, the system was balanced for 50 ps under an NPT ensemble. Finally, molecular dynamics simulations of 35 ns were performed under an NPT ensemble with a time step of 2 fs. We saved trajectory data every 10 ps. The MMGBSA module was used to calculate the binding free energy and to perform energy splitting. All the simulations were performed under periodic boundary conditions using Gromacs 2018 software.

### Capillary electrophoresis

Capillary electrophoresis experiments were performed on a capillary electrophoresis apparatus with UV detection (Cailu Science Apparatus, Beijing, China). The whole length of the fused-silica capillaries was 60 cm with a 50 cm effective length and their inside and outside diameter diameters were 50 μm and 365 μm, respectively. For UV detection, the wavelength was kept at 215 nm. At the beginning of the experiment, the capillary was conditioned with 0.1 M NaOH solution for 5 min, followed by flushing with deionized water for 2 min and PBS for 5 min. Between runs, the capillary was flushed sequentially with 0.1 M NaOH (5 min), deionized water (2 min) and PBS for 5 min to improve the repeatability of the measurements. The compound TG (3 mg) was dissolved in 300 μL NaOH (1 M), and the pH was adjusted to 6.8 via the addition of 425 μL 5% acetonitrile and 7700 μL PBS (20 mM, pH 2.5). After the injection of the sample solution (0.1 mg/mL, 10 cm, 5 s), the inlet of the capillary was moved to a vial containing running buffer, and a 15 kV potential was applied for separation 19.

### Co-immunoprecipitation (CO-IP) assay

Differentiated C2C12 cells were treated with 10 µg/mL TG for 30 min, washed with ice-cold PBS and lysed in cell lysis buffer (25 mM Tris-HCl, 150 mM NaCl, 1 mM EDTA, 1% NP-40, 5% glycerin and protease inhibitor) on ice for 10 min. After centrifugation at 4 ℃, the supernatant was collected. Protein A/G magnetic beads (HY-K0202, MCE, China) were treated according to the manufacturer's instructions, and these beads were incubated with anti-PKACα (1:50; Santa Cruz Biotechnology, CA, USA) or anti-IgG (as control), followed by incubation with supernatant overnight at 4 °C as described in the instructions. The immunoprecipitated proteins were then identified via Western blotting using anti-PKACα and anti-PKAR (1:100; Santa Cruz Biotechnology) antibodies.

### Uptake of 2-NBDG

Briefly, C2C12 myotubes were pretreated with or without 400 μM PA for 24 h to induce insulin resistance, then cells were incubated with TG for 24 h. Then, cells were incubated in Krebs-Ringer phosphate HEPES (KRPH) buffer (118 nM NaCl, 5 mM KCl, 1.2 mM KH_2_PO_4_, 1.3 mM CaCl, 1.2 mM MgSO_4_, 30 mM HEPES, pH 7.4 with 2% BSA) at 37 °C for 30 min, and then treated with 10 μM 2-NBDG (Invitrogen; Thermo Fisher Scientific, Inc.) with or without 100 nM insulin at 37 °C for 1 h in KRPH buffer containing 2% BSA. Following the incubation, cells were washed three times with ice-cold PBS and 2-NBDG uptake into the cells was determined using a fluorescence microscope. The results were quantified using Image J and determined as relative fluorescence intensity with respect to the normal group.

### Animal experiments

Male C57BL/6J mice (4 weeks old, 14-15 g) were purchased from Vital River Laboratory Animal Technology (Beijing, China). Mice were housed in controlled (humidity and temperature) rooms, with free access to food and water throughout the course of the experiments. All mice were anesthetized with isoflurane before sacrifice. The experimental protocols were approved by the Science and Technology Ethics Committee of Northeast Normal University. Animal studies are reported in compliance with the *Interdisciplinary Principles and Guidelines for the Use of Animals in Research, Testing, and Education* by the New York Academy of Sciences, Ad Hoc Animal Research Committee.

For high fat and high sucrose diet (HFHSD)-induced T2DM prevention or treatment experiments, animal management was performed as previously described 11. Briefly, male C57BL/6J mice were randomly divided into five groups of 10 mice each. The ND group was fed a normal diet (ND, 4.5 kcal% fat, Beijing Keao Xieli Feed Co., Ltd.), the HFHSD group was fed a 45 kcal% fat and 20% sucrose diet (HFHSD, Medicience Ltd, Yangzhou, China) and the three TG-treated groups were fed a HFHSD and treated with TG at a concentration of 10 mg/kg, 20 mg/kg or 40 mg/kg, respectively. For the prevention experiment, after one month of HFHSD feeding, mice were administered with different doses of TG every other day by intraperitoneal (i.p.) injection until the fasting blood glucose levels of the HFHSD control mice (i.p. injected with TG solvent) was ≥ 7 mM/L. For the treatment experiment, diabetic mice with fasting blood glucose level higher than 7 mM/L (nearly 4 months of HFHSD feeding) was administered with different doses of TG by i.p. injection every other day for 30 days.

For streptozotocin (STZ)-induced diabetic animal groups, male C57BL/6J mice were fed a HFHSD for 1 month, and were then i.p. injected with STZ (Sigma-Aldrich, MO, USA) dissolved in citric acid-sodium citrate buffer (pH 4.4), for 3 consecutive days at a dose of 30 mg/kg, whereas the ND group was injected with the same vehicle solution. A week later, the fasting blood glucose levels were measured. The diabetic mice were then treated with TG at a concentration of 50 mg/kg, 100 mg/kg or 200 mg/kg or the same volume of solvent (control) via intragastric administration every two day for 1 months. The method refered to the article 20 and has been modified.

For the experiment to knock down PKACα in mouse quadriceps by lentivirus carrying shRNA, male C57BL/6J mice with T2DM induced by 4-month HFHSD feeding were randomly divided into four groups: Lv-shNC, Lv-shNC+40 mg/kg TG, Lv-shPKACα and Lv-shPKACα+40 mg/kg TG. The control Lv-shNC or Lv-shPKACα recombinant lentivirus (the same as those used in C2C12 cells produced by Gene Pharma Co., Ltd.) were multipoint injected into quadriceps of the two hind legs of mice with the same dose (1×10^8^ TU/mL, 250 µL per mouse, more than five injection points). 10 days after lentivirus injection, the mice were administrated with 40 mg/kg TG or the same volume of solvent (vehicle control) by i.p. injection every two days for 1 month.

### IPITT

To perform the intra-peritoneal insulin tolerance test (IPITT), the mice were fasted for 8 h overnight and were then intra-peritoneally injected with insulin at a dose of 0.5 IU/kg body weight. Blood glucose levels were measured before and 15, 30, 45, 60, 90 min after insulin injections. The area under the curve (AUC) of the IPITT results was calculated for each mouse.

### Biochemical analyses of serum

Blood glucose was measured using a glucometer through tail-tip amputation. Serum insulin levels were measured via immunoassays using a mouse ELISA kit (Enzyme-linked Biotechnology, Shanghai, China) according to the manufacturer's protocols. Homeostasis model assessments of insulin resistance (HOMA-IR) 21 were calculated as HOMA-IR = fasting insulin (mIU/L) × fasting blood glucose (mmol/L) / 22.5.

### *Ex vivo* muscle 2-NBDG uptake measurement

The methods for muscle 2-NBDG uptake refered to the article 22 and has been modified. Briefly, muscle samples were quickly excised and pre-incubated in 30 °C baths containing Krebs-Henseleit buffer (KHB) (119 mM NaCl, 4.7 mM KCl, 2.5 mM CaCl_2_, 1.2 mM MgSO_4_, 1.2 mM KH_2_PO_4_, 25 mM NaHCO_3_, pH 7.4) with 0.1% BSA for 1 hour. Muscle samples were incubated in KHB with 10 μM 2-Deoxy-2-[(7-nitro-2,1,3-benzoxadiazol-4-yl) amino]-D-glucose (2-NBDG), stimulated with or without 10 µg/mL TG and 100 nM insulin for 1 hour. The assay was terminated by washing the samples rapidly three times with ice-cold PBS and lysed for 2-NBDG uptake measurement using a FLUOstar OPTIMA system (BMG Labtech, Offenburg, Germany).

### Histopathology

Dissected mouse muscles were fixed in formalin and embedded in paraffin. The paraffin-embedded sections (5 μm) were immunohistochemical (IHC) stained. The sections were deparaffinized and rehydrated, and antigens were retrieved by autoclaving the slides in 10 mM citric acid buffer. The anti-GLUT4 (Cat # sc-53566, 1:100, Santa CRUZ, USA) antibody was used. After washing the slides with PBS, they were treated with a Histostain-Streptavidin-Peroxidase kit (Cat#sp-0024, Bioss, Beijing, China), followed by visualization using a DAB kit (Cat#C02-04001, Bioss, Beijing, China) according to the manufacturer's protocols. The mean optical density (MOD) was analyzed using Image-Pro Plus 5.0 software (Media Cybernetics, Rockville, MD, USA).

### Data and statistical analysis

The data were processed using a two-tailed *t*-test with SPSS software (SPSS Inc., Chicago, USA), and data shown are presented as the mean ± SEM of at least three independent experiments. *** *p* < 0.001, ** *p* < 0.01, and * *p* < 0.05 were considered statistically significant.

## Results

### TG increases GLUT4 expression and promotes glucose uptake in normal and IR C2C12 cells

**Figure 1:**
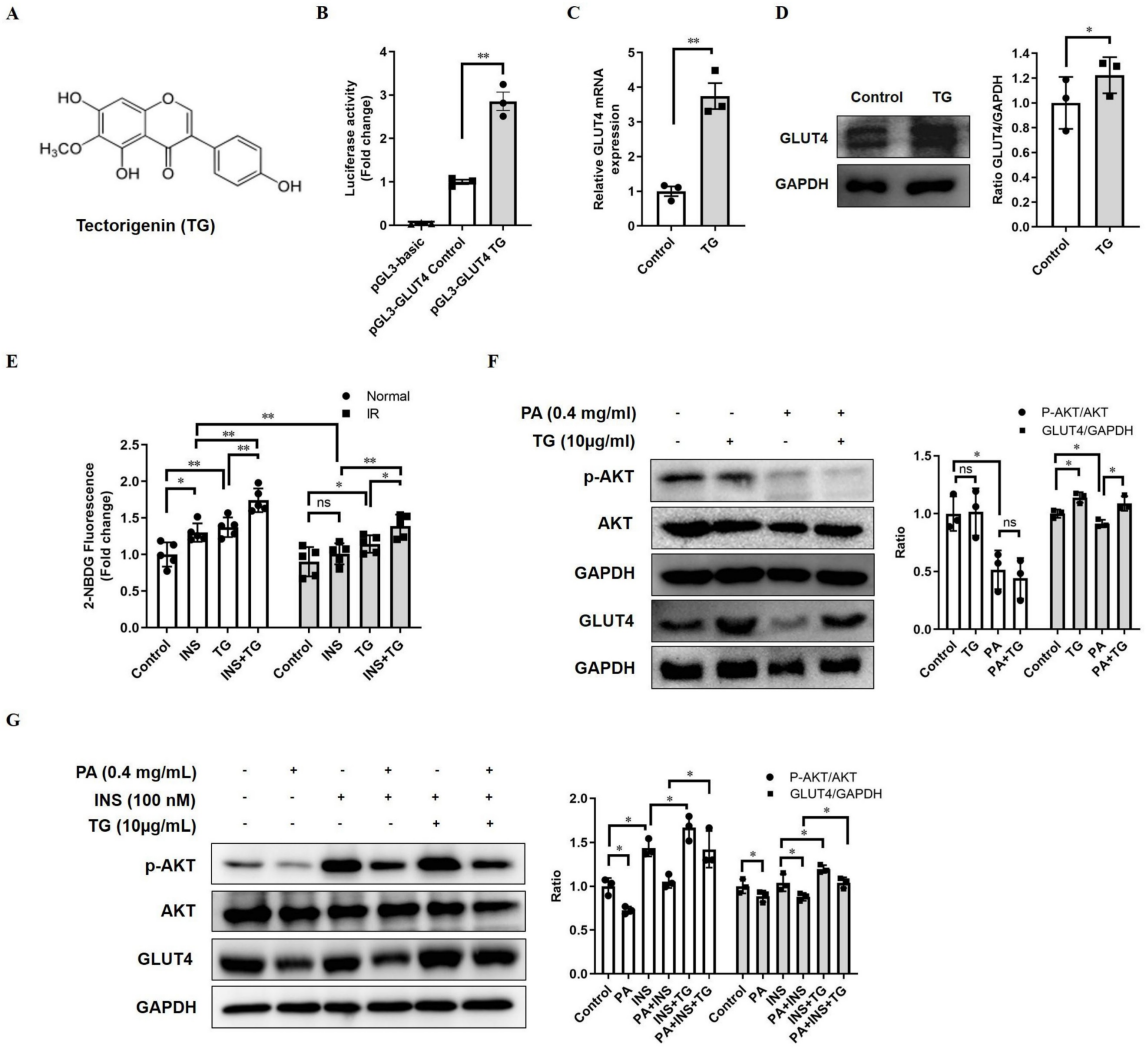
** TG increases GLUT4 expression in C2C12 cells**. (A) The chemical structure of TG. (B) Luciferase activity of PGL3-GLUT4-luc-transfected C2C12 cells treated with or without TG (10 μg/mL) for 24 h. Results are expressed as the fold-induction (over the activity of the control). (C) RT-qPCR analysis of mRNA levels of GLUT4 in C2C12 myotubes, untreated or treated with 10 μg/mL of TG for 24 h. (D) Western blot analysis of GLUT4 protein in C2C12 myotubes, untreated or treated with 10 μg/mL of TG for 24 h. ImageJ software was used for quantitative analysis. (E) Measurement of glucose uptake of C2C12 myotubes using 2-NBDG. C2C12 myotubes were pretreated with 400 μM PA for 24 h to induce IR before 10 μg/mL TG treatment for another 24 h. C2C12 myotubes were then incubated in KRPH buffer at 37 °C for 30 min, followed by incubation in KRPH buffer containing 2% BSA and 10 μM 2-NBDG with or without 100 nM insulin at 37 °C for 1 h. Results were determined based on 2-NBDG relative fluorescence intensity with respect to the normal control group. (F) Western blot analysis of the p-AKT and GLUT4 levels in normal- or PA-induced IR C2C12 myotubes treated with 10 μg/mL TG for 24 h. (G) Western blotting analysis of p-AKT and GLUT4 in PA-induced IR C2C12 myotubes stimulated with 100 nM insulin for 1 h with or without TG 24h-pretreatment. ImageJ was used for quantitative analysis. IR: insulin resistance; INS: insulin; PA: palmitic acid; TG: tectorigenin; ns: not significant; *, *p* < 0.05; **, *p* < 0.01; ***, *p* < 0.001; n ≥ 3.

In view of the crucial roles of GLUT4 in glucose metabolism, which make it a possible drug screening target for IR and T2DM therapy, we established a firefly luciferase reporter screening system, driven by the GLUT4 gene promoter, to screen for potential compounds enhancing GLUT4 expression. The GLUT4 promoter fragment was amplified from a human genomic DNA region and exhibited high activity to drive the expression of luciferase reporter genes when inserted into the pGL3-luc plasmid (pGL3-GLUT4-luc). TG, an O-methylated isoflavone (Fig. 1A) was identified as one of the candidates to significantly increase the luciferase activity of pGL3-GLUT4-luc-transfected cells compared with the control (Fig. 1B), implying that TG may have the potential to promote GLUT4 expression. Then, we further demonstrated that TG treatment increased the mRNA level of GLUT4 in C2C12 myotubes as shown in RT-qPCR analysis (Fig. 1C). Consistently, TG effectively up regulated the protein expression of GLUT4 in C2C12 myotubes as well (Fig. 1D). These results suggested that TG can indeed promote the expression of GLUT4 in skeletal muscle cells, one of the most essential insulin-responsive cells for glucose metabolism.

As GLUT4 is an important glucose transporter in skeletal muscle cells, we next examined whether TG increased the glucose uptake of either normal or palmitic acid (PA)-induced IR C2C12 myotubes with or without insulin stimulation. As shown in Fig. 1E, 24-h TG treatment promoted glucose uptake in normal C2C12 myotubes to a similar degree as that of insulin-induced glucose uptake. Moreover, 24-h TG treatment significantly enhanced the responsiveness of normal C2C12 myotubes glucose uptake to insulin (Fig. 1E). Comparatively, insulin could not effectively induce glucose uptake in PA-induced IR C2C12 myotubes as expected, whereas TG treatment still triggered more glucose uptake to some extent in IR C2C12 myotubes compared to control. Importantly, TG dramatically increased the insulin-induced glucose uptake of IR C2C12 myotubes, indicating an improvement of the insulin-responsiveness of IR myotubes (Fig. 1E). We then demonstrated that TG treatment alone does not affect p-AKT levels in either normal or IR C2C12 myotubes (Fig. 1F), suggesting that TG alone cannot activate the insulin signaling pathway. Simultaneously, we observed an obvious decrease in GLUT4 level in PA-induced IR myotubes, whereas TG partly restored the GLUT4 level of IR myotubes (Fig. 1F). Interestingly, TG treatment increased insulin-induced p-AKT levels in both normal and IR myotubes significantly, although insulin-induced p-AKT levels in IR myotubes were obviously decreased compared with those in normal myotubes (Fig. 1G). As expected, GLUT4 levels in TG-treated C2C12 myotubes were increased compared with TG-untreated counterparts (Fig. 1G). Taken together, these results suggest that TG is a stimulator of GLUT4 expression and it can ameliorate PA-induced IR in C2C12 myotubes by promoting the expression of GLUT4 and potentiating insulin signaling.

### TG upregulates GLUT4 expression through AMPK signaling pathways

**Figure 2:**
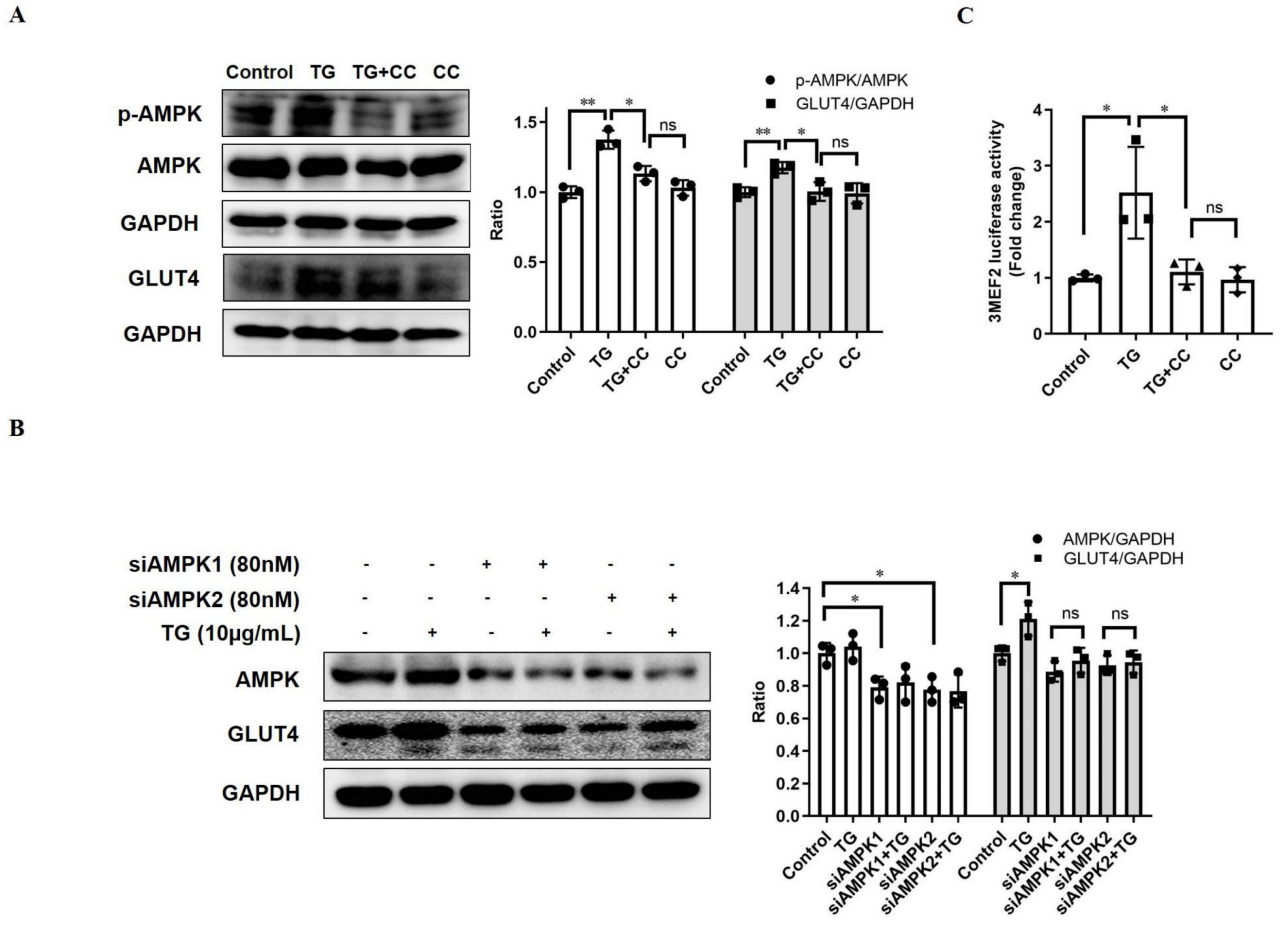
** TG increases GLUT4 expression by activating AMPK/MEF2 signaling.** (A) Western blot analysis of p-AMPK and GLUT4 levels in C2C12 myotubes treated with 10 μg/mL TG, with or without 12.5 µM CC, for 30 min (for p-AMPK) or 24 h (for GLUT4), respectively. (B) C2C12 cells were transfected with AMPK siRNAs, followed by treatment with 10 µg/mL TG for 24 h. The indicated proteins were detected via Western blotting. ImageJ was used for quantification, as shown in the right-hand panels. (C) C2C12 cells were transfected with 3MEF2-luc luciferase reporter constructs before induction to differentiation. After 24 h of treatment of 10 µg/mL TG with or without 12.5 µM CC, C2C12 cells were harvested and subjected to the luciferase activity assay. Values shown represent the mean ± SEM. TG: tectorigenin; CC: compound C; ns: not significant; *, *p* < 0.05; **, *p* < 0.01; ***, *p* < 0.001; n = 3.

AMPK is widely considered as the metabolic master-switch, leading to heightened metabolism 10. Meanwhile, the activation of AMPK upregulates GLUT4 expression by activating MEF2 23. Therefore, we intended to investigate whether TG stimulates GLUT4 expression via the activation of the AMPK/MEF2 pathway. Upon TG treatment, the p-AMPK level in C2C12 cells was markedly increased, indicating that TG could activate AMPK in muscle cells (Fig. 2A). Furthermore, TG-mediated activation of AMPK was abolished by AMPK inhibitor compound C (CC) and, concomitantly, CC treatment completely abolished TG-induced GLUT4 expression at the protein level in C2C12 cells (Fig. 2A). To further confirm the role of AMPK signaling in the effect of TG on GLUT4 expression, we used siRNAs to specifically knock down AMPK levels in C2C12 cells and demonstrated that AMPK knockdown by siRNAs also completely abolished the TG-mediated increase in GLUT4 levels in C2C12 cells (Fig. 2B). Subsequently, we also observed that TG could increase the MEF2 activity-dependent luciferase activity in C2C12 cells by using 3MEF2-luc reporter vectors, implying the activation of MEF2 by TG. However, the increase in MEF2 activity driven by TG was completely eliminated by CC, which indicates that AMPK mediates the activation effect of TG on MEF2 (Fig. 2C). These results suggest that TG enhances the expression of GLUT4 by activating AMPK/MEF2 signaling.

### Prophylactic and therapeutic uses of TG improve insulin sensitivity in HFHSD-induced T2DM mice

**Figure 3:**
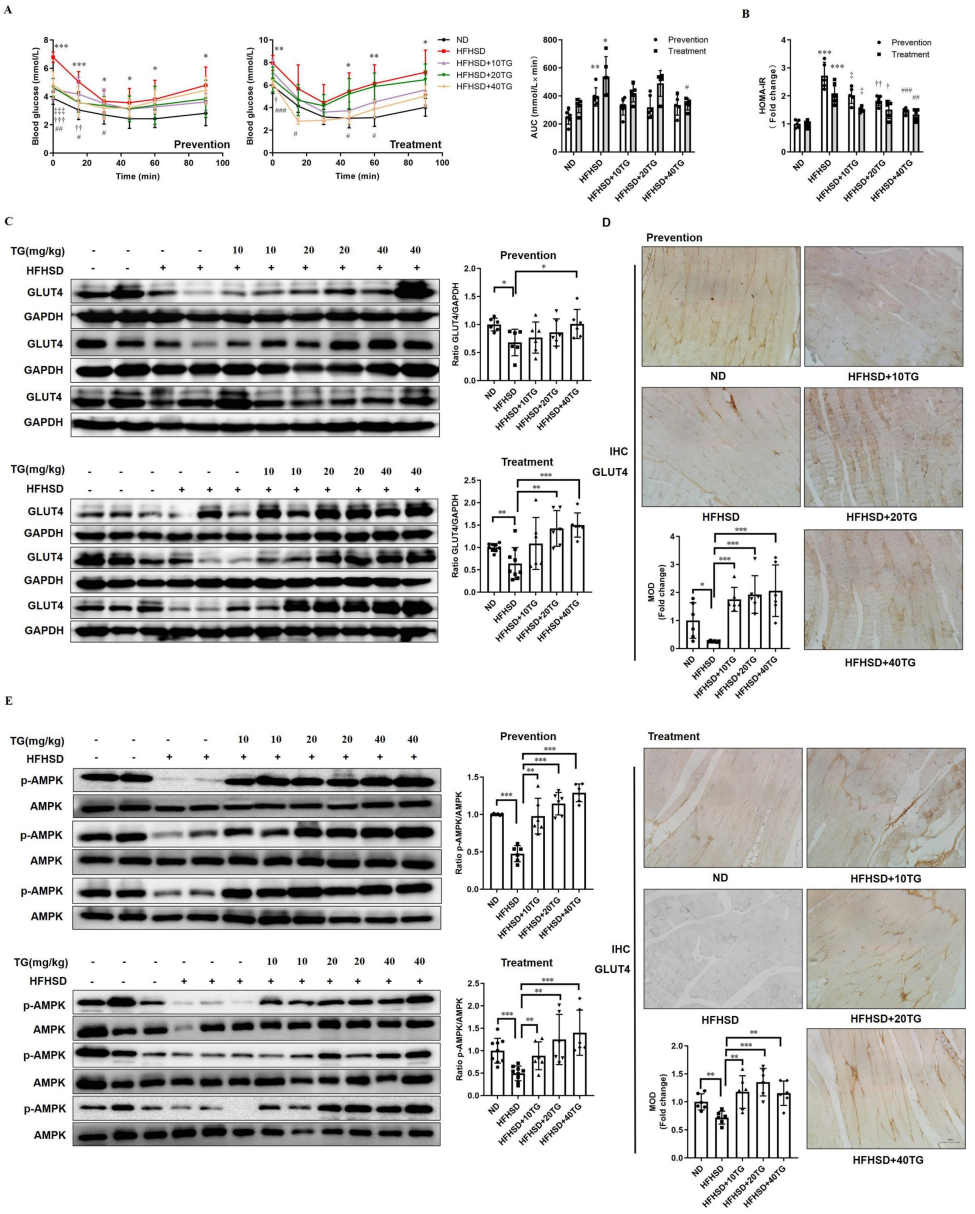
** Prophylactic or therapeutic use of TG ameliorates insulin sensitivity and promotes the GLUT4 expression of muscle tissues in HFHSD-fed mice.** For the prevention experiment, TG was administered every other day for 2 months via an intraperitoneal (i.p.) injection to HFHSD-fed mice starting 1 month after feeding. For the treatment experiment, TG was administered every other day for 1 month via an i.p. injection to mice with fasting blood glucose levels higher than 7mM/L after 4 months of HFHSD feeding. (A) IPITT was evaluated after 8 h fasting. Blood glucose levels of all groups at 0, 15, 30, 45, 60 and 90 min following insulin injection. The area under the curve (AUC) of the IPITT results was analyzed. (B) HOMA-IR of mice. (C) Western blot analysis of GLUT4 expression in muscle tissues from mice. ImageJ software was used for quantitative analysis. (D) Quantification and representative cases of IHC staining for GLUT4 in skeletal muscle in mice. The GLUT4 protein in mouse muscle sections was observed in brown dotted distribution. The mean optical density (MOD) was analyzed using Image-Pro Plus 5.0 software. Bar, 50 μm. (E) The levels of p-AMPK in skeletal muscle were examined via Western blotting. ImageJ software was used for quantitative analysis. ND: normal diet; HFHSD: high-fat and high-sucrose diet; 10TG: 10 mg/kg; TG; 20TG: 20 mg/kg TG; 40TG: 40 mg/kg TG. For Figures A and B: *, p < 0.05 ND versus HFHSD; **, p < 0.01 ND versus HFHSD; ***, p < 0.001 ND versus HFHSD; ‡, p < 0.05, 10TG versus HFHSD; ‡‡‡, p < 0.001, 10TG versus HFHSD; †, p < 0.05, 20TG versus HFHSD; ††, p < 0.01, 20TG versus HFHSD; †††, p < 0.001, 20TG versus HFHSD; #, p < 0.05, 40TG versus HFHSD; ##, p < 0.01, 40TG versus HFHSD; ###, p < 0.001, 40TG versus HFHSD. *, *p* < 0.05; **, *p* < 0.01; ***, *p* < 0.001; n ≥ 5.

The above results showed that TG could increase the expression of GLUT4, induce glucose uptake and improve insulin sensitivity in C2C12 cells *in vitro*. To further explore whether TG has the same beneficial effects *in vivo*, we utilized high fat and high sucrose diet (HFHSD)-induced T2DM mice, which we have used to reveal the hypoglycemic effects of TG previously 11, to assess the effects of TG on GLUT4 expression and IR. Three concentrations of TG were intraperitoneally injected every two days to T2DM mice before or after the onset of hyperglycemia to test for preventive and therapeutic effects, respectively. Intraperitoneal injections for the insulin tolerance test (IPITT) indicated that both prophylactic and therapeutic use of TG displayed potent anti-IR effects and mice administrated with TG showed a decreased area under the curve (AUC) compared with that of HFHSD control mice (Fig. 3A). Consistently, both the prophylactic and therapeutic use of TG dose-dependently decreased homeostasis model assessments of insulin resistance (HOMA-IR), an IR index which is negatively correlative with insulin sensitivity, compared with HFHSD control mice (Fig. 3B). These results suggest that the prophylactic use of TG significantly slowed the development of HFHSD-induced IR in mice and maintained insulin sensitivity, whereas therapeutic use of TG dramatically alleviated IR of T2DM mice and improved insulin sensitivity. Next, Western blotting and immunohistochemical (IHC) analysis were used to detect GLUT4 levels in muscle tissues, respectively. As shown in Fig. 3C and D, the expression levels of GLUT4 significantly declined in the muscle tissues of T2DM mice compared with normal diet (ND)-fed mice. However, prophylactic and therapeutic uses of TG both significantly increased GLUT4 levels in the muscle tissues of T2DM mice in a dose-dependent manner and the GLUT4 levels in the muscles of TG-treated T2DM mice were even higher than those of ND mice (Fig. 3C and D), indicating that TG exhibits strong stimulatory effects on GLUT4 expression in muscle *in vivo*. Furthermore, we examined the p-AMPK levels in muscle tissues to validate the effect of TG on AMPK signaling *in vivo*. As shown in Fig. 3E, the levels of p-AMPK in the skeletal muscle of T2DM mice obviously decreased compared to the ND group, as did GLUT4 levels. Likewise, TG administration, whether for prophylactic or therapeutic purposes, significantly upregulated the levels of p-AMPK in the skeletal muscle of T2DM mice in a dose-dependent manner, and p-AMPK levels in the muscles of T2DM mice administered with medium and high doses of TG were much higher than those of ND mice (Fig. 3E) Taken together, these results suggest that TG can activate AMPK signaling to enhance GLUT4 expression in muscles *in vivo*, which may mediate its anti-IR effects in HFHSD-induced T2DM mice.

### Oral TG treatment increases muscle GLUT4 expression and improves insulin sensitivity in STZ-induced T2DM mice

**Figure 4:**
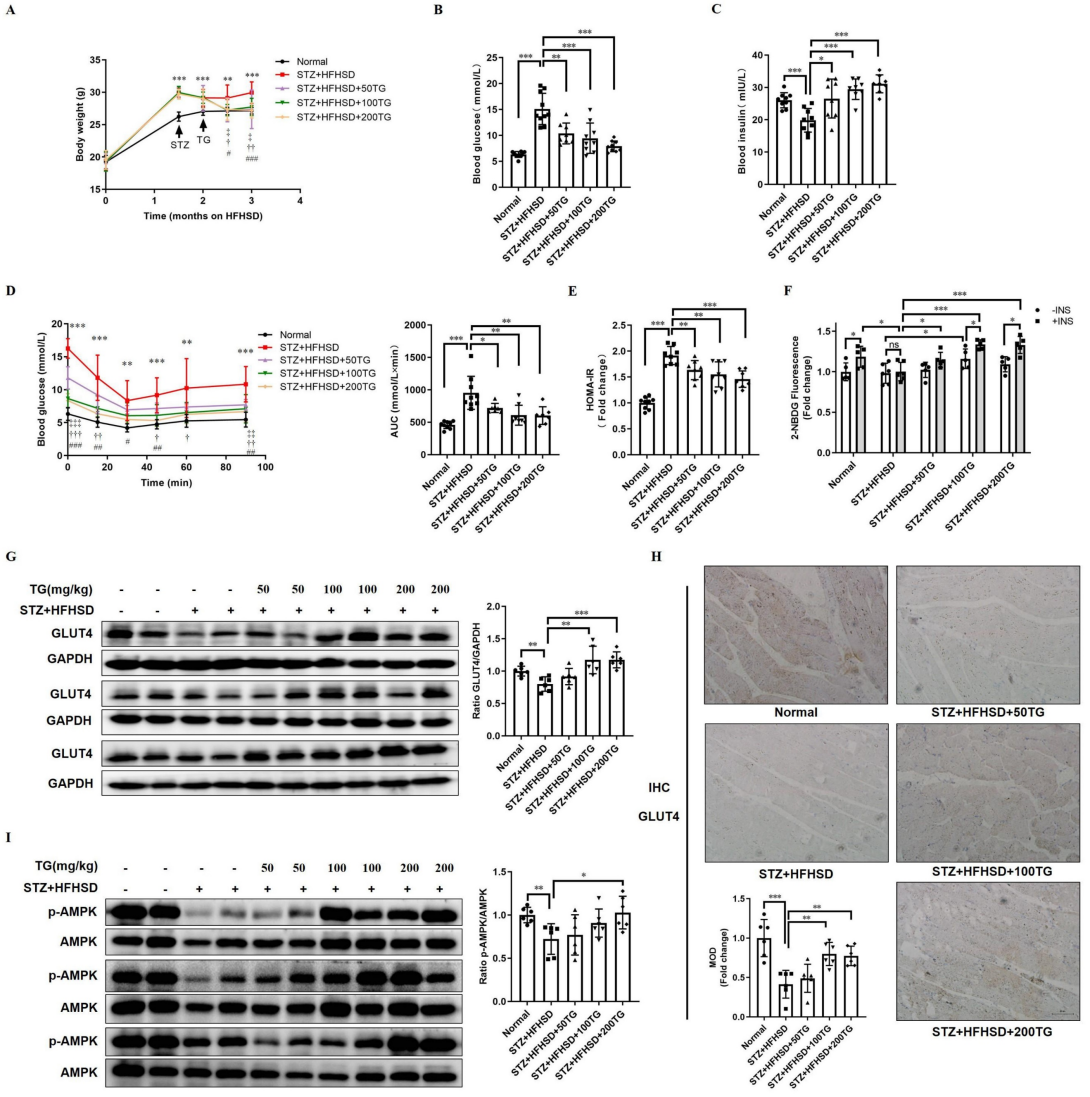
** The therapeutic use of TG ameliorates insulin sensitivity and promotes the GLUT4 expression of muscle tissues in STZ-induced T2DM mice.** To establish STZ-induced diabetic mouse model, mice were fed a HFHSD for 1 month, and then were i.p. injected with STZ for 3 consecutive days at a dose of 30 mg/kg, while the normal control group was simultaneously injected with the same volume of vehicle solution. The diabetic mice were treated with TG or an identical volume of solvent via intragastric administration every two days for 1 months. (A) Fasting body weight of mice. (B) Fasting blood glucose levels. (C) Fasting blood insulin levels. (D) IPITT were evaluated after 8 h of fasting. Blood glucose levels of all groups at 0, 15, 30, 45, 60 and 90 min following insulin injection. The AUC was analyzed. (E) HOMA-IR of mice. (F) Glucose uptake assay of fresh *ex vivo* muscles treated with or without 100 nM insulin *in vitro*. (G) Western blotting analysis of GLUT4 expression in muscle tissues from each group of mice. ImageJ software was used for quantitative analysis. (H) Quantification and representative cases of IHC staining for GLUT4 in skeletal muscle from each group of mice. The MOD was analyzed using Image-Pro Plus 5.0 software. Bar, 50 μm. (I) Western blotting analysis of p-AMPK levels in muscle tissues from each group of mice. ImageJ software was used for quantitative analysis. HFHSD: High fat and high sucrose diet; 50TG: 50 mg/kg TG; 100TG: 100 mg/kg TG; 200TG: 200 mg/kg TG. For broken line graph, **, *p* < 0.01 normal versus STZ+HFHSD; ***, *p* < 0.001 normal versus STZ+HFHSD; ‡, *p* < 0.05, 50TG versus STZ+HFHSD; ‡‡, *p* < 0.01, 50TG versus STZ+HFHSD; ‡‡‡, *p* < 0.001, 50TG versus STZ+HFHSD; †, *p* < 0.05, 100TG versus STZ+HFHSD; ††, *p* < 0.01, 100TG versus STZ+HFHSD; †††, *p* < 0.001, 100TG versus STZ+HFHSD; #, *p* < 0.05, 200TG versus STZ+HFHSD; ##, *p* < 0.01, 200TG versus STZ+HFHSD; ###, *p* < 0.001, 200TG versus STZ+HFHSD. *, *p* < 0.05; **, *p* < 0.01; ***, *p* < 0.001; n ≥ 5.

Though the HFHSD-induced T2DM mouse model closely resembles most human T2DM, the symptoms of this model were relatively mild within the short research period. We established another T2DM mouse model with more severe phenotypes using a low dose of streptozotocin (STZ) damage combined with HFHSD and further assessed the therapeutic effects of the oral administration of TG on IR and T2DM. Different doses of TG were given to STZ-induced T2DM mice via intragastric administration. Compared with T2DM control mice, the levels of body weight and fasting blood glucose significantly declined, whereas fasting insulin levels were obviously elevated in TG-treated groups in a dose-dependent manner (Fig. 4A-C). IPITT demonstrated that TG treatment dramatically and dose-dependently alleviated the severe insulin insensitivity occurring in STZ-induced T2DM mice, indicating the improvement of insulin sensitivity upon TG treatment (Fig. 4D). Consistently, TG intervention decreased the HOMA-IR index of T2DM mice (Fig. 4E). In addition, fresh muscles were isolated from mice of each group and basal or insulin-stimulated glucose uptake *ex vivo* was assessed. As shown in Fig. 4F, the basal glucose uptake level of T2DM mouse muscles was similar to that of normal control, however, under insulin stimulation, normal mouse muscles showed a significant increase in glucose uptake, whereas no increase in glucose uptake was observed in T2DM mouse muscles, indicating the occurrence of insulin resistance in T2DM mouse muscles. By contrast, both the basal and insulin-stimulated glucose uptake of muscles from TG-treated T2DM mice were significantly enhanced compared to T2DM control, especially at the higher doses of 100 mg/kg and 200 mg/kg, for which the glucose uptake levels were even higher than normal control (Fig. 4F). This result demonstrated that TG treatment completely reversed the IR state of T2DM mouse muscles, which may account for the therapeutic effects of TG on systemic IR and diabetes. Furthermore, aberrant reductions in both GLUT4 levels (Fig. 4G-H) and p-AMPK/AMPK ratios (Fig. 4I) in muscles were also observed in STZ-induced T2DM mice, whereas TG treatment increased the GLUT4 expression levels as well as the ratio of p-AMPK/AMPK in the muscles of T2DM mice in a dose-dependent manner (Fig. 4G-I). Altogether, these data further verify that TG can potentiate glucose uptake and improve insulin sensitivity, thus eliciting anti-IR effects by promoting GLUT4 expression via activating the AMPK signalling pathway *in vivo*.

### PKACα is identified as one of the potential targets of TG

**Figure 5:**
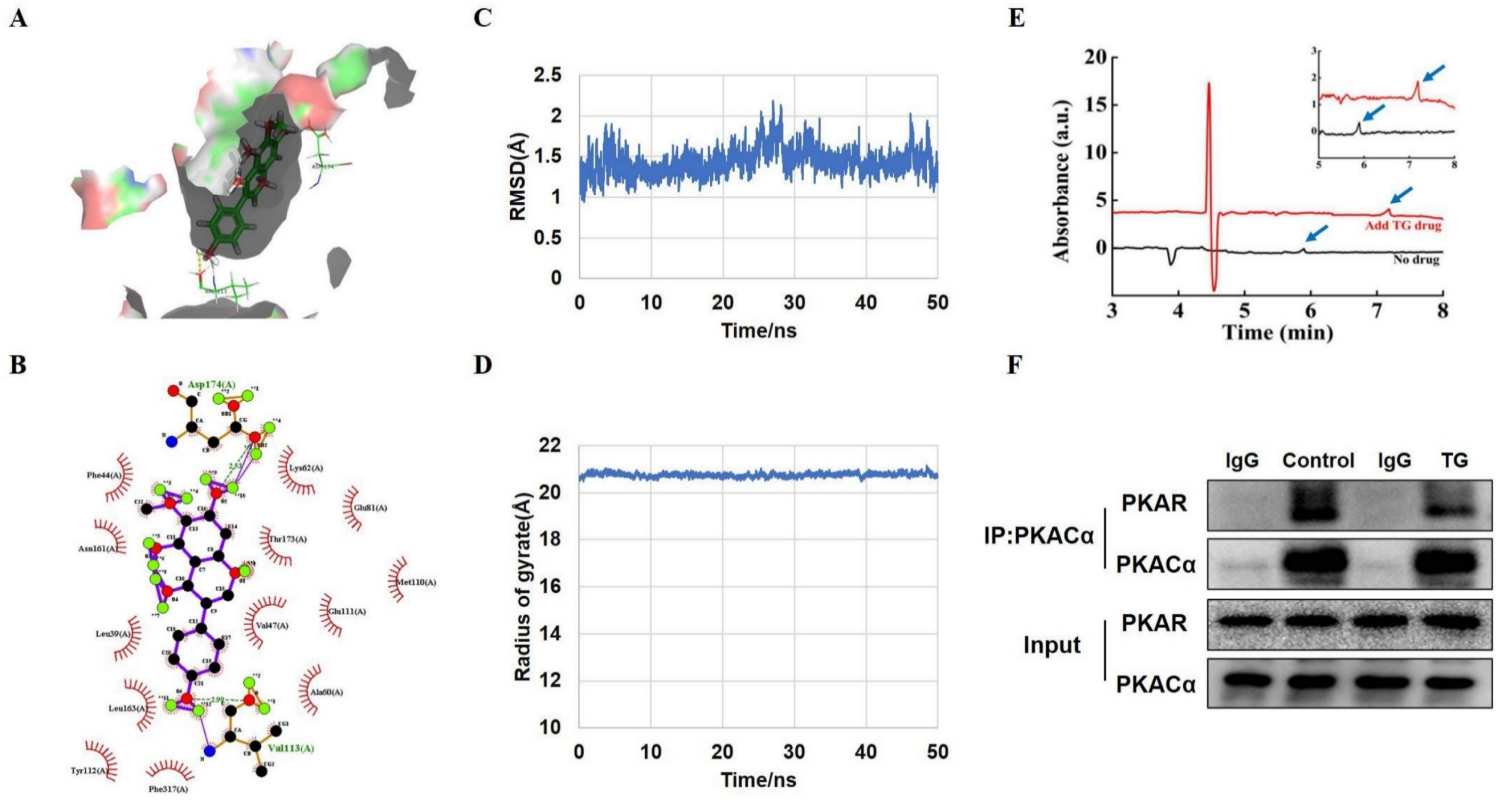
** PKACα is a potential target of TG.** (A) Molecular docking results of the docking poses of TG (CID: 5281811) with the active site of PKACα (PDB: 5UZK). The middle stick model is the TG structure, which is surrounded by the active pocket of PKACα protein. The yellow dashed line is the hydrogen bond formed by TG and PKACα, which is connected to the PKACα-specific amino acid residue stick model. (B) The 2D docked conformation of molecular docking, with the hydrogen bonds represented by green dashed lines and the hydrophobic interaction by arcs using LigPlus. The middle structure is TG, which is surrounded by the amino acid residues that interact with TG. (C, D) Molecular dynamics analysis of the TG-PKACα complex. RMSD curve (C) and radius of gyrate (D). (E) Detection of the direct binding of TG to PKACα via capillary electrophoresis. The black wave line is resulted from the analysis of 0.1 mg/mL PKACα without TG. The red wave line is resulted from the analysis of 0.1 mg/mL PKACα with approximately 0.356 mg/mL TG. The peaks indicated by the blue arrows show that the migration time of the PKACα protein was obviously delayed when TG was added. The image on the top right is an enlarged image. (F) CO-IP analysis of the interaction between PKACα and PKAR in C2C12 cells with or without 10 μg/mL TG treatment.

Next, we intended to identify the potential target by which TG activates AMPK and promotes GLUT4 expression in muscle cells. Molecular docking was first conducted to simulate the interaction of TG with some intracellular kinases related to the activation of AMPK signaling and two docking functions in GOLD 5.2 (GOLD score and ASP score) were used as scoring functions to predict the binding 24. Protein kinase A catalytic subunit alpha (PKACα, PDB: 5UZK), one of the candidate kinases with relatively high scores, displayed high binding affinity for TG (Fig. 5A). The simulation data showed that TG formed hydrogen bonds with Val113 and Asp174 of PKACα and had hydrophobic interactions with Phe44, Asn161, Leu39, Leu163, Tyr112, Phe317, Ala60, Val47, Glu111, Met110, Thr173, Glu81 and Lys62 (Fig. 5B). These hydrogen bonds, coupled with the hydrophobic interactions, contributed to the high stability of the binding.

To further validate the predicted interaction between TG and PKACα, molecular dynamics (MD) simulation was performed to analyze the dynamic binding interactions of the TG-PKACα complex. In order to understand the stability of the molecular dynamics system, we calculated the root mean square deviation (RMSD) curve. As shown in Fig. 5C, the RMSD curve indicates that the conformational changes of PKACα showed a slight fluctuation within the initial 30 ns of the simulation (0-30 ns) and thereafter reached equilibrium (30-50 ns), indicating that TG binding led to a slight conformational change in PKACα but the conformation of the complex rapidly became stabilized. The radius of gyration is the mass-weighted radius, which represents the compactness of the protein molecule. Fig. 5D shows that the gyro radius of the TG-PKACα complex during the kinetic simulation exhibited a slight downward trend in the beginning and then gradually stabilized, indicating no obvious changes in protein compactness. Furthermore, the binding free energy calculation based on the molecular mechanics-generalized born surface area (MM/GBSA) from the MD simulation trajectories (30-50 ns) showed that the binding free energy of TG-PKACα was -28.8 kcal/mol, the van der Waals potential energy was -35 kcal/mol and the electrostatic force was -37 kcal/mol, which are favorable for forming stronger interactions with each other. Altogether, the MD simulation suggested that the interaction between PKACα and TG acquired conformational stability, strongly supporting a high binding affinity of TG to PKACα.

To verify the direct interaction of TG with PKACα, we expressed and purified recombinant PKACα proteins from *E.Coli* 19 and conducted capillary electrophoresis. As shown in Fig. 5E, the migration time of the PKACα protein was obviously delayed when TG was added to the buffer, suggesting that the PKACα proteins displayed good binding with TG. Then we examined the effect of TG binding to the activity of the PKA holoenzyme in the cells to provide intracellular biological evidence for the interaction between TG and PKACα. The PKA holoenzyme is a tetramer assembled from two regulatory (PKAR) and two catalytic subunits. When the PKA holoenzyme is activated, commonly by cAMP binding to each regulatory subunit, the catalytic subunits are released and thereby activated for downstream regulation. 14. Based on this, we performed CO-IP analysis to test the cellular interaction between PKAR and PKACα in C2C12 cells with or without TG treatment. As shown in Fig. 5F, compared with that in control C2C12 cells without TG treatment, much less PKAR was co-immunoprecipitated by anti- PKACα antibodies in TG-treated C2C12 cells, suggesting the release of more PKACα from PKA holoenzyme upon TG treatment. Owning to the above evidence regarding the direct binding of TG to PKACα, we reasonably deduce that TG binding may lead to the separation of PKACα from PKAR in the PKA holoenzyme, implying the activation of PKACα. Taken together, these data indicate PKACα as one of the potential targets of TG. Next, we conducted functional experiments to provide more evidence.

### PKACα is required for TG to promote GLUT4 expression and ameliorate insulin resistance *in vitro*

**Figure 6:**
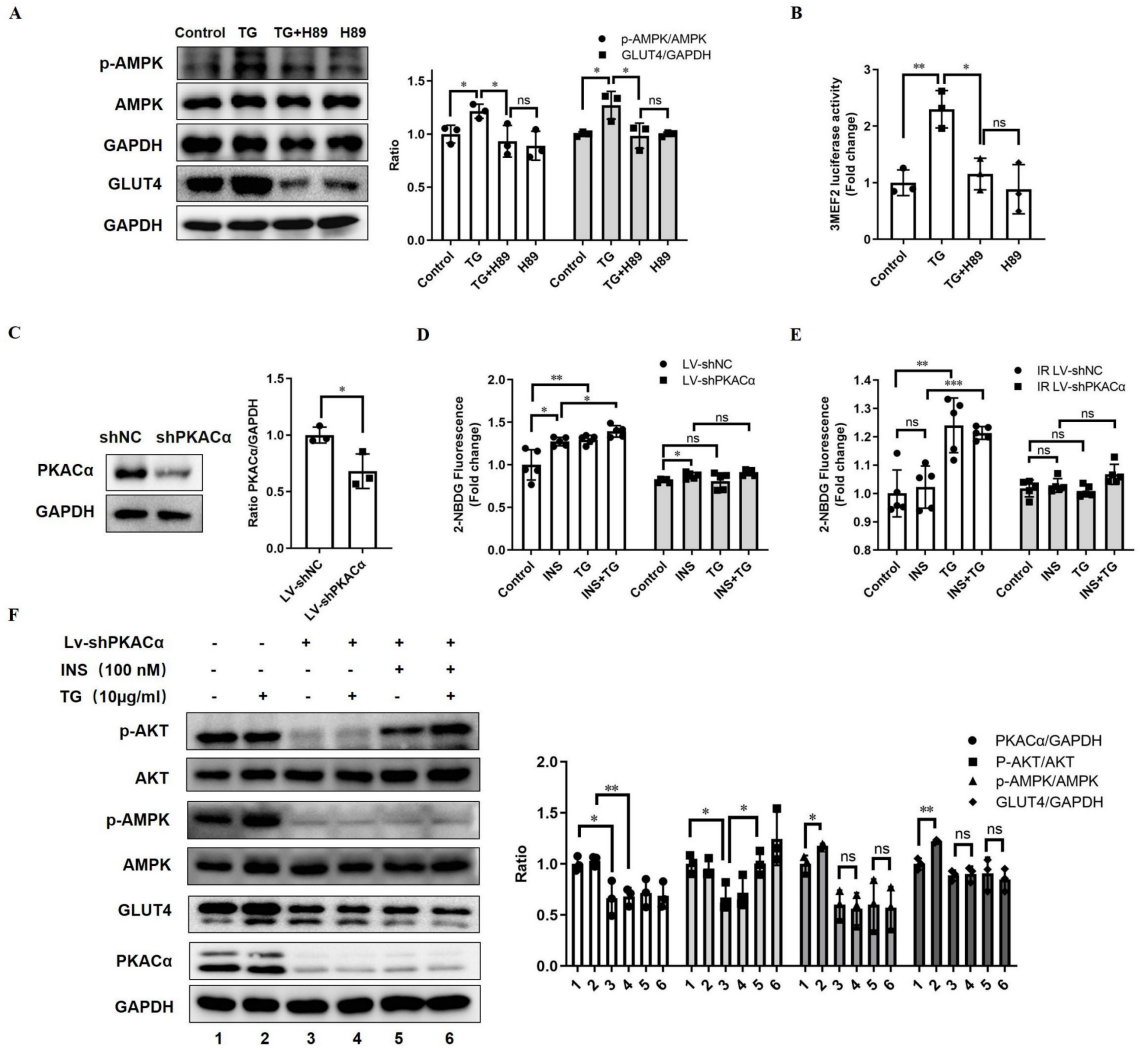
** PKACα is the target of TG, triggering GLUT4 expression and promoting glucose uptake in muscle cells via PKACα/AMPK/MEF2 signaling.** (A) Western blot analysis of GLUT4 levels in C2C12 myotubes treated with 10 µg/mL TG for 24 h, and p-AMPK levels in C2C12 myotubes treated with 10 µg/mL of TG for 30 min, in the presence or absence of 20 µM H89. ImageJ was used for quantification. (B) Reporter assay of C2C12 cells transfected with 3MEF2-luc plasmids, followed by treatment with 10 µg/mL of TG with or without 20 µM H89 for 24 h. (C) Western blot analysis of PKACα in lentivirus-mediated stable PKACα knockdown or control C2C12 myotubes. (D, E) The glucose uptake of stable PKACα-knockdown or control C2C12 myotubes under normal (D) or IR (E) conditions. The C2C12 myotubes were processed in accordance with Figure 1E. (F) Western blot analysis of indicated proteins in the stable PKACα-knockdown or control C2C12 myotubes under insulin stimulation or not. TG: tectorigenin; shNC: C2C12 myotubes with control shRNA; shPKACα: C2C12 myotubes with PKACα knockdown; INS: insulin; IR: insulin resistance; ns: not significant; *, *p* < 0.05; **, *p* < 0.01; ***, *p* < 0.001; n ≥ 3.

To determine whether TG enhances GLUT4 expression and elicits anti-IR effects by targeting and activating PKACα, we first inhibited PKA activity with H89 in C2C12 cells. We observed that the treatment of the PKA inhibitor H89 almost completely abolished the TG-triggered upregulation of GLUT4 and p-AMPK levels (Fig. 6A). In addition, H89 also blocked the TG-mediated enhancement of MEF2 transcriptional activity (Fig. 6B). These results reveal that TG activates AMPK/MEF2 signaling and correspondingly enhances GLUT4 expression by activating PKA.

Next, we established a C2C12 cell line with the stable knockdown of PKACα via lentivirus-mediated shRNA (Lv-shPKACα) (Fig. 6C). In stable control C2C12 cells (Lv-shNC), TG treatment can promote insulin-induced glucose uptake, as well as basal glucose uptake (Fig. 6D), as is consistent with previous observations (Fig. 1E). However, compared with Lv-shNC cells, the basal and insulin-stimulated glucose uptake levels of Lv-shPKACα C2C12 cells were both significantly decreased and insulin responsiveness was also greatly reduced, though a certain degree of increased glucose uptake under insulin stimulation could be still observed in Lv-shPKACα cells (Fig. 6D). More importantly, TG was unable to increase both basal and insulin-stimulated glucose uptake in Lv-shPKACα C2C12 cells (Fig. 6D). Then, we further induced IR in Lv-shPKACα and control Lv-shNC C2C12 cells and found that TG treatment improved the basal and insulin-stimulated glucose uptake of IR Lv-shNC cells (Fig. 6E), as previously shown (Fig. 1E), but this TG-induced improvement disappeared in IR-Lv-shPKACα C2C12 cells (Fig. 6E). These data indicate that PKACα knockdown almost completely abolished the enhancement effects of TG on the glucose uptake of muscle cells, as well as its anti-IR effects.

Moreover, we detected the levels of relevant molecules in Lv-shPKACα C2C12 cells under treatment with TG, insulin or a combination. The results showed that compared with control cells, the levels of GLUT4 and p-AMPK obviously declined following the knockdown of PKACα in Lv-shPKACα C2C12 cells, with or without TG, insulin or combination treatment (Fig. 6F). As anticipated, there was no difference in the levels of GLUT4 or p-AMPK in Lv-shPKACα C2C12 cells with and without TG treatment, indicating that TG lost its effects on GLUT4 and AMPK signaling without PKACα (Fig. 6F). In addition, PKACα knockdown led to a great decrease in the basal p-AKT level shown in Lv-shPKACα C2C12 cells; however, it did not abolish the response to insulin since the p-AKT level increased upon insulin stimulation in Lv-shPKACα C2C12 cells (Fig. 6F). More interestingly, TG treatment could not increase the basal p-AKT level in Lv-shPKACα C2C12 cells as it did in Lv-shNC control cells, but it could still further elevate the insulin-stimulated p-AKT level in Lv-shPKACα C2C12 cells. This result implies that PKACα knockdown does not influence the promotive effect of TG on the insulin sensitivity of muscle cells. Combined with the results presented in Fig. 6D, showing that TG could not promote the insulin-stimulated glucose uptake in Lv-shPKACα C2C12 cells, these data indicate that the TG-triggered elevation of GLUT4 expression is the main mechanism by which TG promotes the basal and insulin-induced glucose uptake of muscle cells, and there are targets other than PKACα for TG to enhance insulin-mediating signaling. In summary, we demonstrated *in vitro* that PKACα is the target of TG, triggering GLUT4 expression and promoting glucose uptake in muscle cells via PKACα/AMPK/MEF2 signaling.

### PKACα is the target for TG to promote GLUT4 expression and ameliorate insulin resistance *in vivo*

Finally, we further functionally validated PKACα as one of the targets of TG *in vivo*. HFHSD-induced type 2 diabetic mice were multi-point intramuscularly injected into the quadriceps of the two hind legs with lentivirus carrying- shRNA targeting PKACα (Lv-shPKACα) or control shRNA (Lv-shNC) and then these mice received 40 mg/kg TG treatment every two days for one month via an i.p. injection, which was the most effective therapeutic plan shown in our previous study (Fig. 3 and 11).

**Figure 7:**
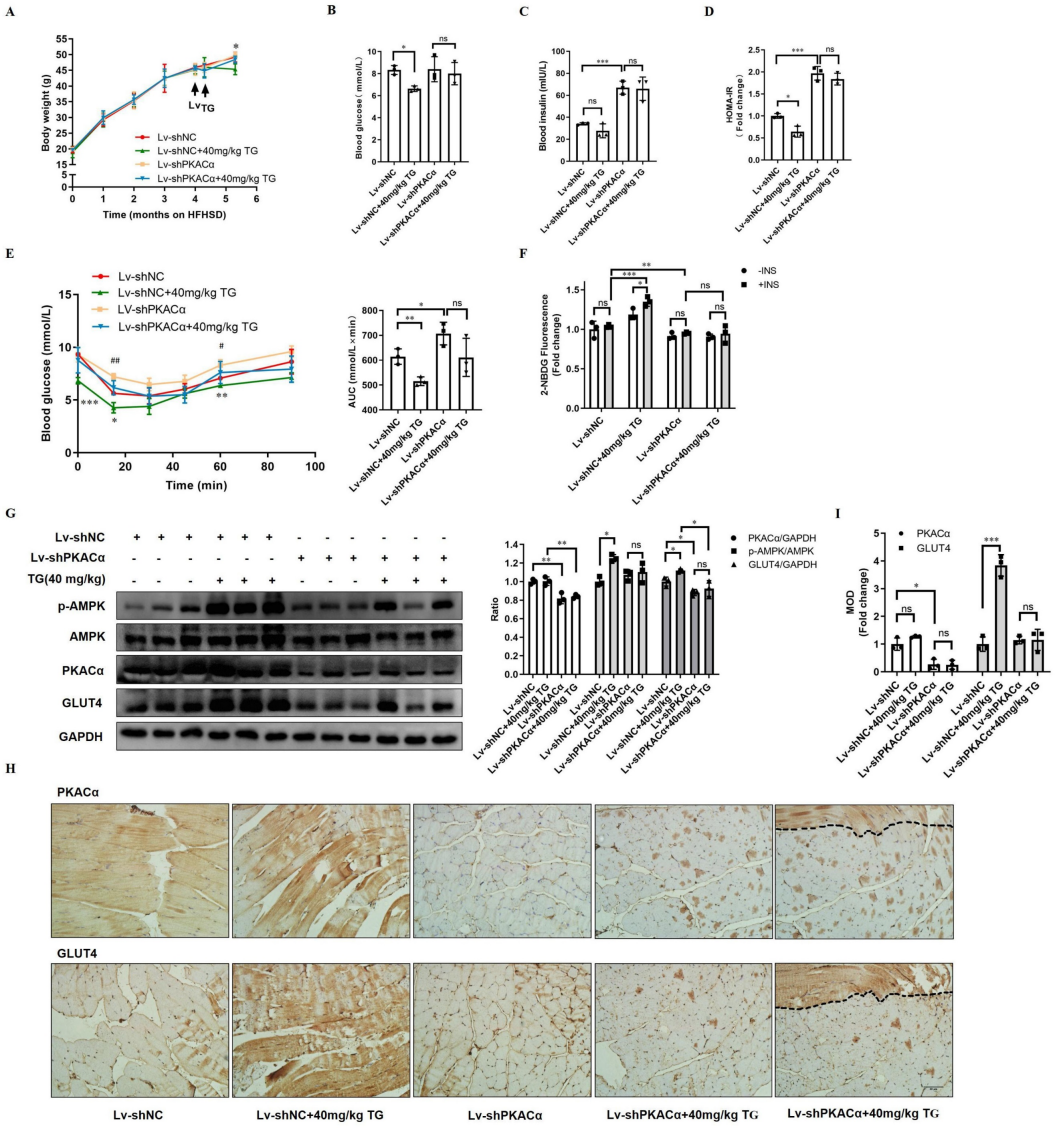
** Therapeutic use of TG ameliorates insulin sensitivity and promotes the GLUT4 expression of muscle tissues in T2DM mice.** HFHSD-induced type 2 diabetic mice were multi-point intramuscularly injected into the quadriceps of the two hind legs with lentivirus-carrying shRNA targeting PKACα (Lv-shPKACα) or control shRNA (Lv-shNC) and then these mice received treatment of 40 mg/kg TG via an i.p. injection every two days for one month. (A) Body weight changes over time. *, *p* < 0.05, Lv-shNC versus Lv-shNC+40 mg/kg TG. (B) Fasting blood glucose levels. (C) Fasting blood insulin levels. (D) HOMA-IR of mice. (E) IPITT were evaluated after 8 h fasting. Blood glucose levels of all groups at 0, 15, 30, 45, 60 and 90 min following insulin injection. The AUC of IPITT was analyzed. *, *p* < 0.05, Lv-shNC versus Lv-shNC+40 mg/kg TG; **, *p* < 0.01, Lv-shNC versus Lv-shNC+40 mg/kg TG; ***, *p* < 0.001, Lv-shNC versus Lv-shNC+40 mg/kg TG; #, *p* < 0.05, Lv-shNC versus Lv-shPKACα; ##, *p* < 0.01, Lv-shNC versus Lv-shPKACα. (F) Glucose uptake of Lv-shNC- or Lv-shPKACα-injected quadriceps from each group of mice. (G) Western blot analysis of PKACα, p-AMPK and GLUT4 levels in Lv-shNC- or Lv-shPKACα-injected quadriceps from each group of mice. ImageJ software was used for quantitative analysis. (H) Representative IHC staining for PKACα and GLUT4 of Lv-shNC- or Lv-shPKACα-injected quadriceps from each group of mice. Notably, the most right-handed slide for PKACα or GLUT4 staining respectively include a strip of muscle (the field above the dotted line) which remained a normal level of PKACα (top) indicating without Lv-shPKACα infection in this strip of muscle, while the field below the dotted line showed a low level of PKACα as expected. (I) IHC quantification and the MOD was analyzed using Image-Pro Plus 5.0 software. Bar, 50 μm. ns: not significant. *, *p* < 0.05; **, *p* < 0.01; ***, *p* < 0.001; n = 3.

Systemically, on one hand, Lv-shPKACα mice without TG treatment exhibited no significant differences in body weight and fasting blood glucose level; however, they exhibited a sharp increase in fasting blood insulin level and HOMA-IR index compared with the Lv-shNC mice (Fig. 7A-D). Consistently, IPITT results also showed that Lv-shPKACα mice displayed more severe insulin intolerance compared with Lv-shNC mice (Fig. 7E). These data suggest that the knockdown of PKACα expression only in parts of muscles can also aggravate the insulin resistance of diabetic mice, but hyperglycemia may not worsen temporarily, owing to compensatory hyperinsulinemia. On the other hand, TG treatment has much less of a therapeutic effect on Lv-shPKACα diabetic mice compared with its effects on Lv-shNC diabetic mice. TG treatment did not lower the body weight, fasting blood glucose levels, blood insulin levels or the HOMA-IR index of Lv-shPKACα mice (Fig. 7A-D). As for the IPITT results, the AUC showed a decreasing trend but did not reach statistical significance in TG-treated Lv-shPKACα mice when compared with untreated Lv-shPKACα mice (Fig. 7E). The above data indicate that the loss of PKACα expression in parts of muscles almost completely abolished the systemic therapeutic effects of TG on diabetes, which was totally beyond our expectation. We originally anticipated that only the regional muscles with Lv-shPKACα virus injection would lose their responsiveness to TG treatment, namely no enhancement of GLUT4 expression and glucose uptake; but the general therapeutic effects of TG would be affected to a lesser degree. Therefore, these results imply that the therapeutic effects of TG on IR and T2DM are closely related with its role in muscle glucose metabolism, with PKACα as one of its essential targets.

Next, the regional quadriceps multi-point injected with Lv-shPKACα or Lv-shNC lentivirus were isolated from each mouse for further examination. Fig. 7F showed that the basal glucose uptake level of Lv-shPKACα-injected muscles was lower than that of Lv-shNC-injected muscles either under TG treatment or not and both Lv-shPKACα- and Lv-shNC-injected muscles from TG-untreated mice did not respond to insulin stimulation, showing an IR state as expected. While both basal and insulin-stimulated glucose uptake were significantly higher in Lv-shNC-injected muscles from TG-treated mice than those from untreated mice, both the basal and insulin-stimulated glucose uptake of Lv-shPKACα-injected muscles displayed no difference between TG-treated and untreated mice (Fig. 7F). This result demonstrates that TG-stimulated glucose uptake in muscles depends on PKACα.

Then, the muscle tissues were subjected to Western blotting and IHC staining analysis. The efficient knockdown of PKACα in Lv-shPKACα-injected muscles from either TG-treated or untreated mice was verified when compared with Lv-shNC-injected muscles (Fig. 7G-I). Following the downregulation of PKACα, the levels of p-AMPK and GLUT4 did not significantly increase in Lv-shPKACα-injected muscles from TG-treated mice compared with those from untreated mice (Fig. 7G-I). Comparatively, a significant increase in the levels of p-AMPK and GLUT4 were observed in Lv-shNC-injected muscles from TG-treated mice compared with those from untreated mice, which is consistent with previous data (Fig. 3). More interestingly, two serial sections of the same Lv-shPKACα-injected muscles from TG-treated mice (the rightest slice sections in Fig 7H) exceptionally contained a strip of muscle (the field above on the dotted line) which remained a normal level of PKACα due to a lack of virus infection (top section). Then, we observed a strong GLUT4 staining only in this strip of muscle (the same field above the dotted line in the bottom section) to a degree similar to that in Lv-shNC-injected muscles from TG-treated mice, suggesting that this strip of muscle expressed higher level of GLUT4 in response to TG treatment which is in sharp contrast to other parts of this section with PKACα knockdown (Fig. 7 H). These results further validate that TG enhances the expression of GLUT4 in muscles via PKACα-mediated AMPK activation and correspondingly promotes the glucose uptake of muscles and ameliorates IR and T2DM.

## Discussion

IR has attracted a great deal of attention in recent years, and it is crucial to prevent the onset of IR or develop a timely and effective therapy to slow or stop IR progression for the prevention and treatment of T2DM and other diseases included in metabolism syndrome. Generally, anti-IR treatment focuses on enhancing insulin signaling. However, our study concentrates on improving GLUT4 expression, which is usually downregulated in T2DM patients 4. The main insulin-responsive glucose transporter, GLUT4, is a key regulator of systemic glucose homeostasis and plays an important role in the pathophysiological process of IR. Therefore, GLUT4 is considered to be one of the important protein molecules that control IR and T2DM. Here, we illuminated the pharmacologic effects of the natural compound TG on the enhancement of GLUT4 expression in skeletal muscles, by which TG dramatically alleviates IR and T2DM. More importantly, we elucidated PKACα as one of the targets of TG and uncovered the mechanism underlying this process as well. This study provides theoretical and experimental basis for the development of TG as a drug for the treatment of IR and T2DM in the future.

The skeletal muscle is the largest organ in the body by mass, responsible for 80% of postprandial glucose uptake from the circulation 25. Therefore, skeletal muscle is the main target organ for glucose metabolism in peripheral tissues and the occurrence of IR in skeletal muscle may substantially decrease the capacity of glucose metabolism of the body. Here, we show that TG can enhance glucose uptake in skeletal muscle and improve IR both *in vivo* and *in vitro* via promoting GLUT4 expression, but this is possibly irrelevant to stimulating insulin signaling.

In both normal and IR-C2C12 cells, TG could promote the expression of GLUT4 and increase the basal and insulin-stimulated glucose uptake, whereas TG alone could not activate AKT signaling but could enhance insulin-stimulated AKT signaling during co-treatment with insulin (Fig. 1). This result implies that the improvement effects of TG on basal glucose uptake are mediated mainly by elevating the GLUT4 level, whereas the elevation of the GLUT4 level and the improvement of insulin signaling may both account for the enhancement of insulin-stimulated glucose uptake by TG in normal and IR-C2C12 cells. However, when we knocked down PKACα in C2C12 cells, TG treatment did not promote GLUT4 expression, nor did it increase insulin-stimulated glucose uptake, although it still enhanced insulin-stimulated AKT signaling (Fig. 6). This result confirms that the improvement effects of TG on glucose uptake and IR in C2C12 cells is mainly mediated by the promotion of GLUT4 expression. In addition, in T2DM mice, we observed that TG displayed significant hypoglycemic and anti-IR effects and augmented the GLUT4 levels of skeletal muscles as well. Nevertheless, multiple mechanisms might be involved in TG's therapeutic effects on T2DM *in vivo* other than its effects on skeletal muscle. For example, it is more likely that TG may promote GLUT4 expression and ameliorate IR in adipose or other insulin target tissues; moreover, we have previously shown that TG can protect islet β-cells against glucotoxicity and lipotoxicity *in vitro* and *in vivo* and thus plays a role in maintaining β-cell mass 11. Moreover, TG has been shown to exhibit anti-inflammatory activity and regulate adipogenic differentiation and adipocytokines 13. However, when lentivirus was only injected into quadriceps of the two hind legs of T2DM mice to locally knock down PKACα expression, TG treatment almost completely lost its therapeutic effects on T2DM. These results further validate the notion that skeletal muscle, as the principal site of insulin-stimulated glucose uptake, is also considered the primary driver of whole-body insulin resistance 2. More importantly, our study demonstrates that improvement of the GLUT4 expression level in skeletal muscle is extraordinarily effective in the alleviation of IR.

To our knowledge, there are several reports related to TG pharmacokinetics *in vivo* 26-28. For example, Zhang et al 27 shown that TG achieved the maximum plasma concentration within 0-0.5 h after oral administration, suggesting that the entrance of TG into blood was rapid through absorbance in the stomach, while TG was eliminated after 12 h in plasma which may be due to lower concentration (only 23.0 mg/kg) and an extensive metabolism. In addition, Shi et al 28 reported the urinary excretion of TG after oral administration to rats at dose of 65 and 130 mg/kg. Urinary excretion of drug largely determines the intensity and duration of drug action, and is generally used to examine biological effects 28. The excretion rates of TG reached a maximum between 12 and 24h and TG remained detectable in urinary excretion from 0-72 hours after oral administration at 130 mg/kg (the higher dose) 28, indicating that the higher doses of TG, the longer of duration of TG to act. The tissue distribution of TG *in vivo* has not been reported yet. In our study, the doses of TG by oral administration to mice were 50 mg/kg, 100 mg/kg or 200 mg/kg. According to the reports from Zhang et al and Shi et al, we believe that there was a considerable quantity of TG in the plasma which retained for enough time to elicit biological activity in mice. Besides, our study demonstrated that the skeletal muscle from the mice with TG administration showed increased expression of GLUT4, higher levels of phospho-AMPK and enhanced capability of glucose uptake. All these effects are the same as those of TG on C2C12 cells *in vitro*. Therefore, we presume that TG can be distributed into skeletal muscle through blood stream to elicit these effects. In addition, Shi et al 28 showed that totally 26 metabolites were detected in rat urine after oral administration of TG. Therefore, we can't exclude the possibility that some of the metabolites of TG may also exert the same pharmacological effects as TG *in vivo*.

TG is a natural compound with multiple pharmacological activities. Here, we identify PKACα as one of the drug targets of TG. TG binds directly with PKACα, which results in the dissociation of PKACα from the regulatory subunits of PKA which may result in the subsequent complete activation of PKA. Activated PKA then may lead to the phosphorylation of AMPK, which eventually activates MEF2, triggering GLUT4 transcription. Through this mechanism, TG dramatically enhances GLUT4 expression in skeletal muscle, by which it elicits its preventive and therapeutic effects on IR and T2DM. The PKA-mediated signaling system plays a central role in the regulation of the energy balance and metabolism by integrating multiple systems, including the neural system, as well as peripheral organs such as adipose tissue, the liver, the pancreas, etc. 14. We demonstrated that hypericin, another PKACα agonist, can be used to prevent and treat non-alcoholic fatty liver 19 and suggested PKACα as an important target for the regulation of liver lipid metabolism. Similarly, our study (data not shown) also observed the therapeutic effects of TG on HFHS-induced hepatic steatosis. Moreover, we found that TG can obviously reduce the body weight of HFHS-fed mice, and similar results were also observed in hypericin-treated HFHS-fed mice 19. Interestingly, Dickson 29 showed that mice expressing an activated PKA catalytic subunit in adipose tissue (Adipoq-caPKA mice) were protected from high-fat-diet-induced weight gain, accompanied by an improvement in adipose tissue health and general metabolic health. Therefore, we presume that TG may also exert the above beneficial effects on hepatic steatosis and obesity by targeting PKACα, which is worth further investigation. PKA signaling is also involved in regulating cell bioenergetics and survival in diabetes 15. Therefore, one of the key aspects of our future studies will be whether TG's protective effect on islet β-cells, which we observed previously 11, is also mediated by targeting and activating PKA.

In addition, aside from the regulation of metabolism, PKA signaling plays important roles in other physiological and pathological processes. For example, PKA is involved in heart functions, including contraction, metabolism, ion fluxes and gene transcription, and abnormal PKA activity is likely to cause the progression to cardiomyopathy and heart failure. Therefore, PKA is also considered to be a potential target for the development of heart failure drugs 30. TG, through activation of PKA, may have the potential to be applied in treating heart failure or other diseases owing to defective PKA signaling. On the other hand, it is noteworthy that TG's structure is simple and it is prone to bind with target proteins. Therefore, TG is likely to be a multi-target compound with versatile pharmacologic activities, which needs to be revealed through future in-depth studies.

In summary, we identified the natural compound TG to enhance the expression of GLUT4 and further demonstrated its preventive and therapeutic roles against IR and T2DM both *in vitro* and *in vivo*. Moreover, for the first time, we identified PKACα as one of the drug targets of TG and revealed that TG exerts its pharmacological effects against IR at least partly through binding with PKACα to activate the PKA/AMPK pathway and promote GLUT4 expression. This study not only supports TG as a new drug candidate to treat IR and its related diseases, but also enriches our knowledge of PKA signaling in glucose metabolism in skeletal muscle.

## Figures and Tables

**Table 1:** Primers

Primers	5′-3′
*GLUT4* promoter region	sense: CGCTCGAGGCAAAGGAATCAAGAAGGGATGTAA
	antisense: GCAAGCTTTGAATCCTACTTCGGAGCCTATCTG
*Glut4*	sense: TCCTTCTATTTGCCGTCCTC
	antisense: GGTTTCACCTCCTGCTCTAA
*Gapdh*	sense: AAATGGTGAAGGTCGGTGTG
	antisense: TGAAGGGGTCGTTGATGG

**Table 2:** Interfering RNA sequences

siRNA	siNC	sense: 5′-UUCUCCGAACGUGUCACGUTT-3′
		antisense: 5′-ACGUGACACGUUCGGAGAATT-3′
	siAMPK-1	sense: 5′-GAGGAGAGCUAUUUGAUUATT-3′
		antisense: 5′-UAAUCAAAUAGCUCUCCUCTT-3′
	siAMPK-2	sense: 5′-GCGUGUACGAAGGAAGAAUTT-3′
		antisense: 5′-AUUCUUCCUUCGUACACGCTT-3′
shRNA	shPKACα	CCTTCAAGGACAACTCAAA
	shNC	TTCTCCGAACGTGTCACGT

## Abbreviations

IR: insulin resistance
T2DM: type 2 diabetes
TG: tectorigenin
GLUT4: glucose transporter protein 4
MEF2: myocyte enhancer factor 2
AMPK: AMP-activated protein kinase
CaMK: calmodulin-dependent protein kinase
PKA: protein kinase A
PKACα: catalytic subunit α of PKA
PA: palmitic acid
HFHSD: high fat and high sucrose diet
ND: normal diet
STZ: streptozotocin
IPITT: intra-peritoneal insulin tolerance test
AUC: area under the curve
IHC: immunohistochemical
MOD: mean optical density
HOMA-IR: homeostasis model assessments of insulin resistance
MD: molecular dynamics
RMSD: root mean square deviation
MM/GBSA: molecular mechanics-generalized born surface area
PKAR: regulatory subunit of PKA
CO-IP: co-immunoprecipitation
